# Glucocorticoid Insensitivity in Virally Infected Airway Epithelial Cells Is Dependent on Transforming Growth Factor-β Activity

**DOI:** 10.1371/journal.ppat.1006138

**Published:** 2017-01-03

**Authors:** Yuxiu C. Xia, Asmaa Radwan, Christine R. Keenan, Shenna Y. Langenbach, Meina Li, Danica Radojicic, Sarah L. Londrigan, Rosa C. Gualano, Alastair G. Stewart

**Affiliations:** 1 Lung Health Research Centre, Department of Pharmacology & Therapeutics, The University of Melbourne, Parkville, Victoria, Australia; 2 Department of Microbiology and Immunology, The University of Melbourne at the Peter Doherty Institute for Infection and Immunity, Melbourne, Victoria, Australia; Thomas Jefferson University, UNITED STATES

## Abstract

Asthma and chronic obstructive pulmonary disease (COPD) exacerbations are commonly associated with respiratory syncytial virus (RSV), rhinovirus (RV) and influenza A virus (IAV) infection. The ensuing airway inflammation is resistant to the anti-inflammatory actions of glucocorticoids (GCs). Viral infection elicits transforming growth factor-β (TGF-β) activity, a growth factor we have previously shown to impair GC action in human airway epithelial cells through the activation of activin-like kinase 5 (ALK5), the type 1 receptor of TGF-β. In the current study, we examine the contribution of TGF-β activity to the GC-resistance caused by viral infection. We demonstrate that viral infection of human bronchial epithelial cells with RSV, RV or IAV impairs GC anti-inflammatory action. Poly(I:C), a synthetic analog of double-stranded RNA, also impairs GC activity. Both viral infection and poly(I:C) increase TGF-β expression and activity. Importantly, the GC impairment was attenuated by the selective ALK5 (TGFβRI) inhibitor, SB431542 and prevented by the therapeutic agent, tranilast, which reduced TGF-β activity associated with viral infection. This study shows for the first time that viral-induced glucocorticoid-insensitivity is partially mediated by activation of endogenous TGF-β.

## Author Summary

In this study, we investigate how respiratory viral infection interferes with the anti-inflammatory actions of glucocorticoid (GC) drugs, which are a highly effective group of anti-inflammatory agents widely used in the treatment of chronic inflammatory airway diseases, including asthma and chronic obstructive pulmonary disease (COPD). Exacerbations of both asthma (“asthma attacks”) and COPD are often caused by viral infection, which does not respond well to GC therapy. Patients are often hospitalized placing a large burden on healthcare systems around the world, with the young, elderly, and those with a poor immune system particularly at risk. We show that viral infection of airway epithelial cells causes increased expression and activity of transforming growth factor-beta (TGF-β), which interferes with GC drug action. Importantly, we have shown for the first time that inhibiting TGF-β activity in the airways could serve as a new strategy to prevent and/or treat viral exacerbations of chronic airway diseases.

## Introduction

Exacerbations of asthma and chronic obstructive pulmonary disease (COPD) are commonly associated with airway viral infection, including respiratory syncytial virus (RSV), human rhinovirus (RV) and influenza A virus (IAV) [1, 2]. RSV infection is a major cause of acute respiratory disease (i.e. bronchiolitis), especially in infants and the elderly [3–5]. Most children are infected by RSV at least once by 2 years of age [3]. RSV infection in children does not elicit long-term immunity, and the adaptive immunity following natural infection is poorly protective even in adults. Thus, re-infection occurs throughout life, even with the identical RSV strain [6, 7]. Severe RSV infection in infancy may result in Th2 and Th17-biased responses, that influence allergic airway inflammation[6]. Approximately 50% of the children who had severe RSV bronchiolitis were subsequently diagnosed with asthma [8, 9]. In addition to RSV, RV and IAV are also commonly detected in patients with asthma and COPD exacerbations [2, 10, 11].

During cellular infection, the viral pathogen-associated molecular patterns (PAMPs), such as viral single-stranded (ss) RNA, double-stranded (ds) RNA, dsRNA-like structures (panhandles), the 5’ triphosphate structure of viral RNA or some unidentified RNA structures, are detected by pattern recognition receptors, including toll-like receptors (TLRs), retinoic acid-inducible gene (RIG)-1-like receptors (RLRs), and nucleotide-binding oligomerization domain (NOD)-like receptors (NLRs) [1, 12–14]. Activation of these innate immune receptors induces secretion of primary anti-viral mediators, including Type-I and -III interferons (IFN-α/β and IFN-λ) to combat the viral infection [12]. Simultaneously, respiratory viral infection induces the secretion of an array of other pro-inflammatory cytokines and chemokines to recruit inflammatory cells to the site of infection to facilitate viral clearance. The infiltrating inflammatory cells also release inflammatory mediators that may induce tissue damage and compromise function [12, 15].

There is no effective therapeutic strategy for either RSV or RV infection except for supportive care, including hydration and oxygenation. Development of effective vaccines is challenging due to the immature infant immune system in early-life RSV infection, and to the large number of RV serotypes [5, 16, 17]. The approved antiviral drugs for the treatment of IAV, the M2 ion channel blocker (amantadine and rimantadine) and neuraminidase inhibitors (zanamivir, oseltamivir and peramivir), are associated with adverse effects or have limited efficacy, respectively. The IAV vaccine is updated annually; however, it still gives limited protection [18]. The most commonly used anti-inflammatory drugs for asthma and COPD exacerbations are glucocorticoids (GCs). However, the majority of clinical studies have found that respiratory viral infection responds inadequately to the anti-inflammatory actions of either inhaled or systemic GCs [19–25]. Moreover, the effect of GCs on virus-induced cytokine secretion is controversial. GCs have been shown to inhibit RSV infection-induced interleukin (IL)-8 and macrophage inflammatory protein (MIP-1) secretion from neutrophils [26], and IL-11 production by lung fibroblasts [27]. However, GCs have been shown to have no effect on the RSV-induced release of IL-8 and MIP-1 during infection of either Hep-2 epithelial cells or primary bronchial epithelial cells [28]. The mechanism by which the inflammation associated with respiratory viral infection is unresponsive to GCs treatment remains unclear.

Airway epithelium is a key target for both GC activity and GC resistance [29, 30]. Infection with RSV or RV has been shown to impair GC transactivation in alveolar epithelial A549 cells, bronchial epithelial BEAS-2B cells, and in submerged primary bronchial epithelial cells [31–36]. However, there is limited understanding of the underlying mechanism of viral-induced GC resistance in epithelial cells. Upon viral infection, airway epithelial cells produce an array of pro- and anti-inflammatory cytokines and chemokines including the type-I and -III interferons (IFN-α/β and IFN-λ), TNFα, IL-4, IL-8, IL-13, IL-17, CCL3 and RANTES [37], some of which have been shown to interfere with GC action in epithelial cells: TNFα inhibits GC transactivation in A549 cells and BEAS-2B cells [38]; IL-17 induces GC insensitivity in the human bronchial epithelial cell line, 16HBE14o- [39]; and, IFN-λ-induced JAK/STAT signaling activation is insensitive to GC action in A549 cells and air-liquid interface (ALI) differentiated primary human bronchial epithelial cells (HBECs) [40]. Viral infection of airway epithelial cells with RSV [41], RV [42, 43], or IAV [44, 45] also results in increased expression, secretion, and activity of the pleiotropic growth factor transforming growth factor-β (TGF-β). Moreover, endogenous TGF-β enhances RSV replication by induction of cell cycle arrest in an autocrine manner [41], and increases RV replication by suppression of type I/III IFN expression [42, 43]. Importantly, our group recently found that TGF-β causes a profound impairment of GC activity in A549 cells, BEAS-2B cells and in ALI-HBECs [46, 47].

We therefore hypothesize in the current study that viral-infection induced glucocorticoid insensitivity in epithelial cells is due to activation of endogenous TGF-β. We provide evidence that autocrine activation of TGF-β mediates the GC insensitivity induced by RSV, RV, and IAV infection in epithelial cells. Moreover, we also examined the anti-allergic agent tranilast, which has been widely used in Japan and South Korea [48, 49]. Tranilast has therapeutic effects in many conditions including inflammation, renal fibrosis, autoimmune disorders and cancer. It has been reported that tranilast inhibits the expression and activity of TGF-β in different cell types [50–52]. We show that tranilast inhibits the expression and activity of TGF-β in epithelial cells, and provide the first evidence that TGF-β modulators may be suitable novel therapeutics to restore sensitivity to GC actions during viral infection.

## Results

### RSV Infection Impairs Glucocorticoid Transactivation

**Fig 1:**
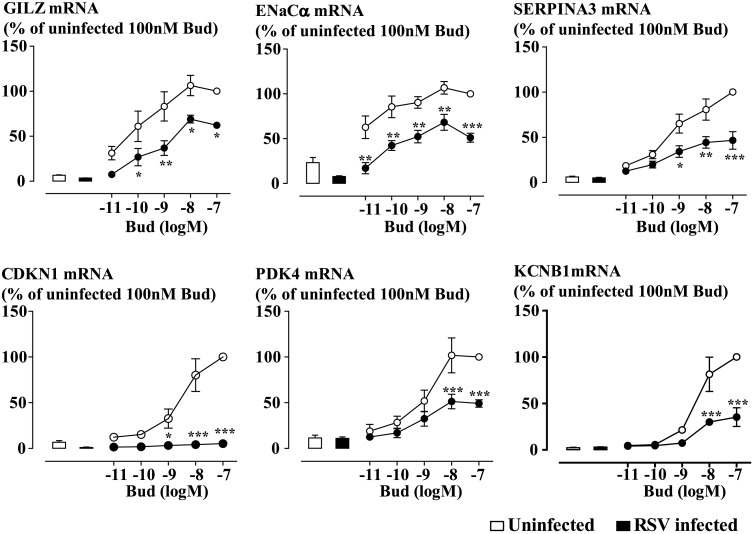
RSV infection impairs budesonide-induced gene expression. BEAS-2B cells were infected with RSV at MOI 0.1 for 48 hours. Budesonide (Bud) was added for the last 4 hours. Total RNA was extracted and analyzed by RT-qPCR. Gene expression is expressed as a percentage of 100nM Bud level in uninfected cells. Data are presented as mean ± SEM, n = 3–4 independent experiments. *P<0.05, **P<0.01, ***P<0.001.

**Fig 2:**
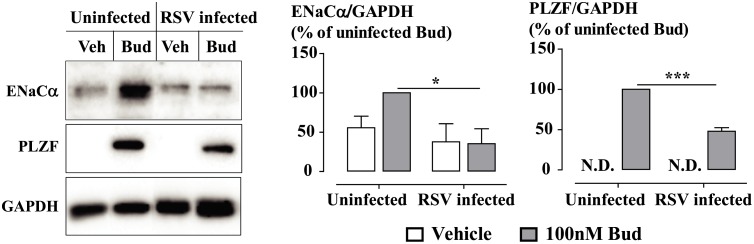
RSV infection impairs budesonide-induced protein expression. BEAS-2B cells were infected with RSV at MOI 0.1 for 48 hours. Budesonide (100nM, Bud) was added for the last 24 hours. Expression of ENaCα and PLZF protein was analyzed by Western blotting. The membrane was then stripped and re-probed for GAPDH expression for normalization. Protein expression was expressed as a percentage of 100nM Bud response in uninfected cells. Densitometry data are presented as mean ± SEM, n = 3 independent experiments. *P<0.05, ***P<0.001, Bud in RSV infected cells *c*.*f*. Bud in uninfected cells.

Budesonide (0.01-100nM) induced a concentration-dependent increase in the expression of the selected GC-inducible genes. The expression of most of the genes assessed was markedly impaired by RSV infection at a multiplicity of infection (MOI) of 0.1 virus units/cell for 48 hours (Fig 1). Genes impaired in this manner included those encoding glucocorticoid-inducible leucine zipper (GILZ), which is an anti-inflammatory/anti-proliferative gene; epithelial sodium channel-α subunit (ENaCα), which regulates the airway fluid levels by absorbing Na+ ions; α-1 antichymotrypsin (SERPINA3), which inhibits the activity of proteases; cyclin-dependent kinase inhibitor 1C (CDKN1C), which is a cell cycle negative regulator; pyruvate dehydrogenase kinase isozyme 4 (PDK4), which decreases glycolytic metabolism; and the potassium channel shab-related subfamily B member 1 (KCNB1), which regulates epithelial electrolyte transport. We examined the effect of viral infection on maximum GC response; therefore 100nM budesonide was used for the following gene expression experiments based on the concentration-response curves. Suppression of budesonide-induced glucocorticoid response element (GRE) activity was also observed in BEAS-2B cells infected with MOI 0.1 RSV for 48 hours (S1 Fig). Budesonide at 1nM was used for the GRE activity study, as we previously showed that GRE activity requires lower concentrations of GCs to reach the maximum response compare to the concentrations for GC-inducible gene expression. Although RSV infection did not influence cell viability, it decreased the total cell number (S2(A) Fig). The intracellular expression of RSV A2 strain N gene mRNA, measured as an index of viral load, was unaffected by budesonide (S3 Fig). The expression of GC-inducible proteins ENaCα and the promyelocytic leukemia zinc finger (PLZF, a transcriptional repressor in control of cell proliferation and differentiation) was also examined. We found that RSV infection clearly impaired the expression of budesonide-induced ENaCα and PLZF protein (Fig 2).

### RSV Infection Impairs Pretreated Glucocorticoid Transactivation

**Fig 3:**
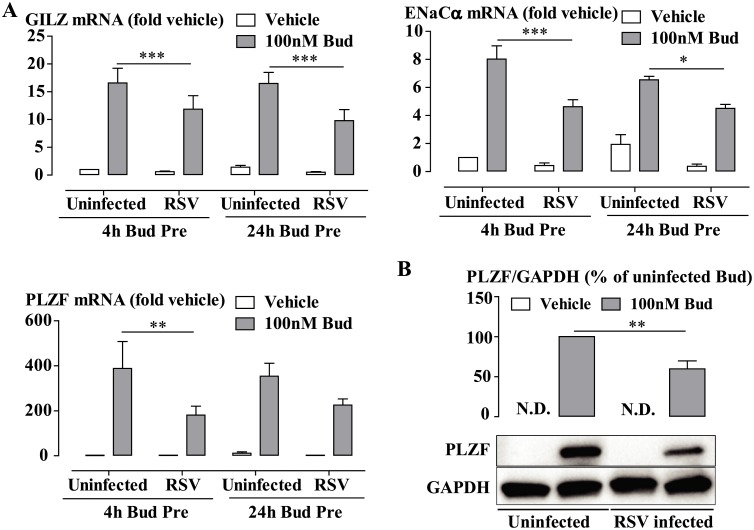
RSV infection impairs budesonide-induced genes and protein expression in budesonide-pretreated cells. (A) BEAS-2B cells were treated with/without budesonide (Bud, 100 nM) either 24 hours or 4 hours prior to RSV infection. Bud was re-added into the cells after 1 hour inoculation of RSV at MOI0.1. Total RNA was extracted after 48 hours, and analyzed by RT-qPCR. Gene expression is expressed as fold change from uninfected vehicle-treated cells. Data are presented as mean ± SEM, n = 4 independent experiments. *P<0.05, **P<0.01, ***P<0.001. (B) BEAS-2B cells were pretreated with 100nM Bud for 4 hours prior to RSV infection for 48 hours. Cell lysates were collected and PLZF protein expression was analyzed by Western blotting. Densitometry data are presented as mean ± SEM, n = 4 independent experiments. **P<0.01.

As respiratory viral infection is a major trigger of exacerbations of asthma or COPD, we therefore investigated the effect of RSV infection in BEAS-2B cells, which had been pretreated with budesonide for 24 hours or 4 hours, to emulate the sequence of exposure for asthma or COPD patients who are on GC therapy at the time of viral infection. We found that budesonide-induced expression of GILZ, ENaCα and PLZF mRNA was significantly impaired by subsequent RSV infection. Moreover, budesonide-induced expression of PLZF protein was significantly reduced by subsequent RSV infection (Fig 3).

### RSV Infection-Induced Impairment of Glucocorticoid Action Is Associated with Activation of ALK5

**Fig 4:**
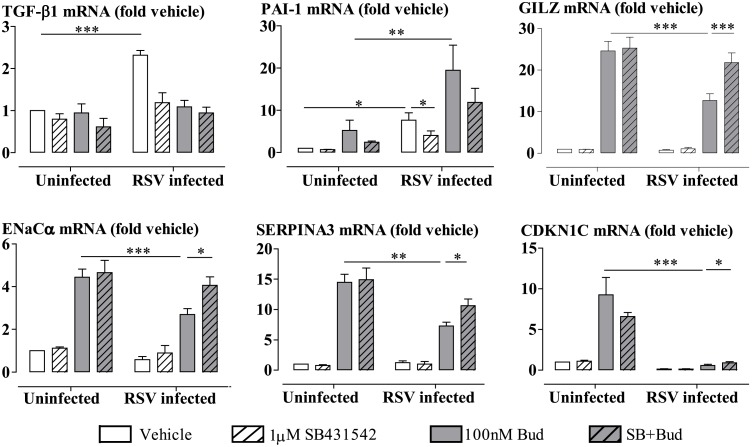
SB431542 prevents RSV impairment of budesonide-induced transactivation. BEAS-2B cells were pre-incubated with SB431542 (1μM) for 30 min prior to RSV infection at MOI 0.1 for 48 hours. Budesonide (Bud) was added for the last 4 hours. Total RNA was extracted and gene expression was measured by RT-qPCR. Gene expression is expressed as fold change from uninfected cells treated with vehicle. Data are presented as mean ± SEM, n = 3–6 independent experiments. *P<0.05, **P<0.01, ***P<0.001.

RSV infection significantly increased the expression of TGF-β1 mRNA in BEAS-2B cells (Fig 4). The cells were pre-incubated for 30 min with the TGF-β receptor (activin-like kinase 5 (ALK5/ TGFβR1)) selective inhibitor, SB431542 (1μM using concentration validated in previous studies [46, 47]) to ascertain the activity of the endogenous TGF-β [53]. TGF-β-inducible gene PAI-1 (plasminogen activator inhibitor-1) was used as a consistent marker for TGF-β activity. Inhibition of ALK5 attenuated the RSV-induced mRNA expression of PAI-1 in BEAS-2B cells [54]. Moreover, PAI-1 can also be induced by GC in different cell types [55, 56]. We found, as expected, that budesonide (100 nM) significantly induced the expression of PAI-1 mRNA, and further enhanced the induction by RSV infection (Fig 4).

TGF-β impairs glucocorticoid function in both bronchial epithelial cells (BEAS-2B cell line and primary ALI-HBECs) and pulmonary epithelial cells (A549 cell line) [46, 47]. The impairment of glucocorticoid action in these cell types was found to be dependent on activation of the TGF-β receptor kinase ALK5 [46, 47]. Inhibition of ALK5 using SB431542 (1μM) completely prevented RSV infection—impaired GRE activation in BEAS-2B cells (S1 Fig). Moreover, inhibition of ALK5 using SB431542 completely prevented or significantly attenuated the RSV infection (48 hours)-induced impairment of budesonide-induced expression of GILZ, ENaCα and SERPINA3, with little effect on CDKN1C expression levels (Fig 2). However, SB431542 did not influence the total cell number (S2B Fig), or the intracellular expression of RSV A2 strain N gene (S3A Fig). Similar findings were obtained by pretreating the cells with a structurally distinct ALK5 inhibitor GW788388 (1μM) [53] (S4 Fig). In addition, transfection of ALK5-targeted siRNA resulted in more than 50% knockdown of ALK5 protein expression (S5A Fig). A concomitant impairment of ALK5 activity was confirmed by measurement of TGF-β-induced phosphorylation of Smad2, which is a critical downstream signaling molecule for TGF-β/ALK pathway (S5B Fig). Importantly, transfection of ALK5 siRNA showed similar effects to the ALK5 inhibitors in restoring GC sensitivity.

### RV Infection, IAV Infection and Poly(I:C) Stimulation Impairs Glucocorticoid Action, Whilst Poly(I:C)(HMW)/LyoVec Stimulation Has No Effect

**Fig 5:**
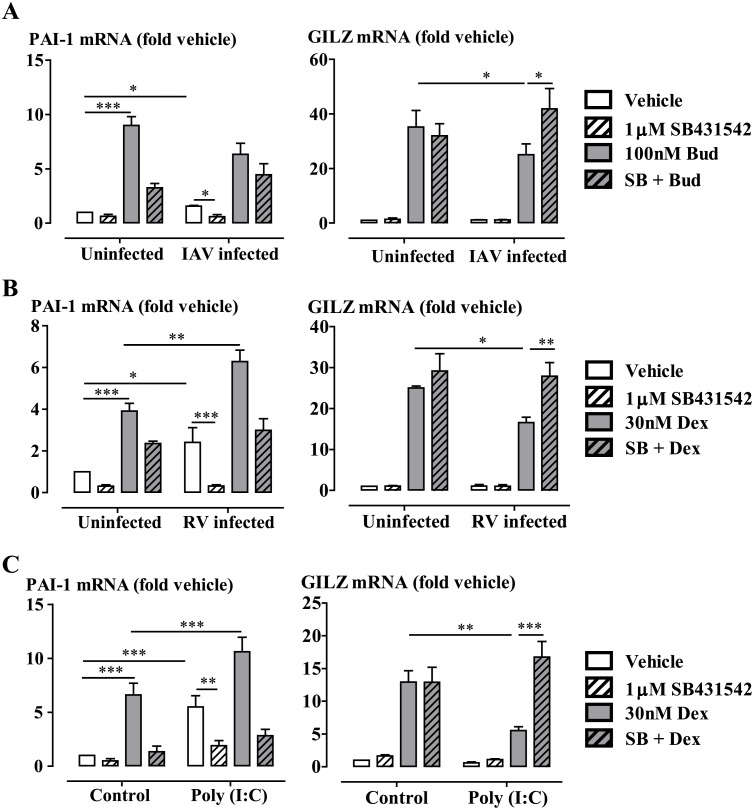
ALK5 activation is required for impairment of budesonide (Bud) or dexamethasone (Dex) activity by IAV infection (A), RV infection (B) and Poly(I:C) stimulation (C). BEAS-2B cells were pre-incubated with SB431542 (1μM) for 30 min prior to **(A)** IAV (MOI 0.1) or **(B)** RV (MOI 1) infection for 48 hours, or **(C)** poly(I:C) stimulation (10 μg/ml) for 24 hours. Budesonide (Bud) was added for the last 4 hours. Gene expression is expressed as fold change from the level in uninfected vehicle-treated cells. Data are presented as mean ± SEM, n = 4 **(A and C)** and n = 3 **(B)** independent experiments. *P<0.05, **P<0.01, ***P<0.001.

Infection of BEAS-2B cells with IAV at MOI 0.1 (Fig 5A), RV at MOI 1 (Fig 5B) for 48 hours, or treatment of the cells with Poly(I:C) (10 μg/ml) for 24 hours (Fig 5C), impaired budesonide (100nM) or dexamethasone (30nM)-induced GILZ expression. Inhibition of ALK5 using SB431542 (1μM) prevented IAV, RV or Poly(I:C) impairment of GC-induced GILZ expression. Moreover, inhibition of ALK5 attenuated the IAV, RV or Poly(I:C)-induced PAI-1 mRNA expression Again, budesonide (100 nM) significantly increased PAI-1 expression (Fig 5). However, treatment of BEAS-2B cells with RLR (RIG-1 and MDA-5) ligands Poly(I:C)(HMW)/LyoVec (0.01–1μg/ml) had no effect on dexamethasone (30nM)-induced GILZ and ENaCα expression (S6 Fig).

### RSV Infection Induces TGF-β Expression and Activity via TLR3-ERK1/2 Pathway

**Fig 6:**
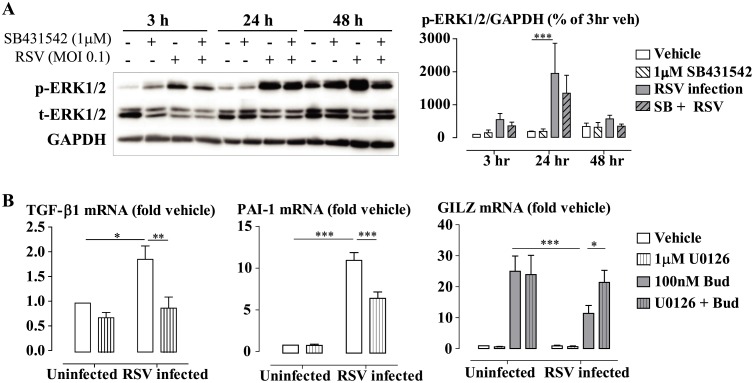
RSV infection-induced TGF-β production is associated with ERK1/2 phosphorylation. **(A)** BEAS-2B cells were pre-incubated with SB431542 (1μM) for 30 min prior to RSV infection at MOI 0.1 for 3 hours, 24 hours and 48 hours. Densitometry data are presented as mean ± SEM of 3 experiments. ***P<0.05, RSV infection *c*.*f*. vehicle. **(B**) BEAS-2B cells were pre-incubated with U0126 (1 μM) for 30 min prior to RSV infection. Budesonide (Bud) was added for the last 4 hours. Gene expression is expressed as fold change from the uninfected vehicle-treated cells. Data are presented as mean ± SEM, n = 3 independent experiments. *P<0.05, ***P<0.001.

RSV infection is known to activate a variety of intracellular signaling cascades. A number of the underlying kinases are involved in non-canonical TGF-β signaling pathways, including p38^MAPK^, ERK1/2, JNK, Akt and NFkB. We found that RSV infection induced the phosphorylation of ERK1/2 kinase in BEAS-2B cells, and pretreatment of the cells with SB431542 showed a trend to decrease the phosphorylation of ERK1/2 (Fig 6A). We therefore further investigated the involvement of the ERK1/2 kinase using the MEK1/2 inhibitor U0126 at 1 μM, at concentration validated in previous studies [46, 47]. Pre-incubation of the cells with U0126 (1μM) reduced the induction of TGF-β and PAI-1 mRNA during RSV infection and attenuated the RSV infection impairment of budesonide-induced expression of GILZ mRNA (Fig 6B).

**Fig 7:**
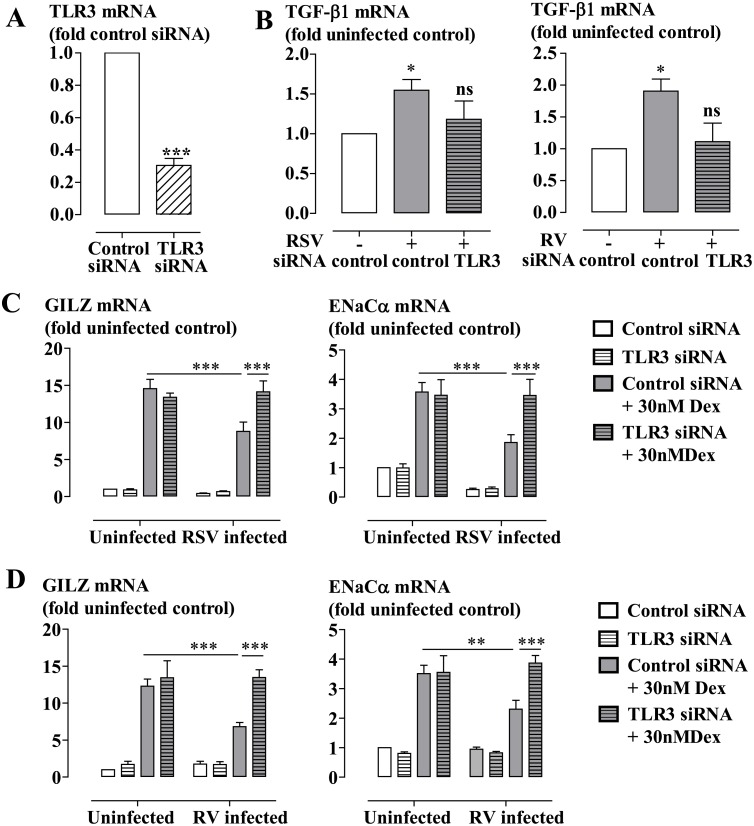
Impairment of glucocorticoid-induced gene expression with viral infection is mediated by activation of TLR3. BEAS-2Bcells transfected with TLR3 targeted siRNA were infected with RSV for 44 hours prior to the addition of dexamethasone (30nM, Dex) or vehicle for 4 hours. TLR3 siRNA reduced TLR3 mRNA expression **(A)**, prevented viral infection (RSV or RV)-induced TGF-β expression **(B**), and attenuated RSV **(C)** or RV **(D)**-impaired Dex-induced gene expression. Data are presented as mean ± SEM for n = 4–5 independent experiments. *P<0.05, **P<0.01 ***P<0.001.

Stimulation of BEAS-2B cells with the TLR3 ligand Poly(I:C) (10 μg/ml) also induced EKR1/2 phosphorylation (S7 Fig). We next examined whether the viral infection-impaired GC action was mediated by activation of TLR3 using targeted siRNA. Transfection of TLR3-targeted siRNA induced a knockdown of approximately 70%, which was stable throughout the experimental period (72 hours) (Fig 7A). We found that knockdown of TLR3 largely inhibited both RSV and RV-induced TGF-β expression (Fig 7B), and prevented the viral infection impairment of dexamethasone-induced gene expression (Fig 7C and 7D).

### RSV Infection-Induced Impairment of Glucocorticoid Action Is Not Dependent on Impaired GRα Expression and/or Localization

**Fig 8:**
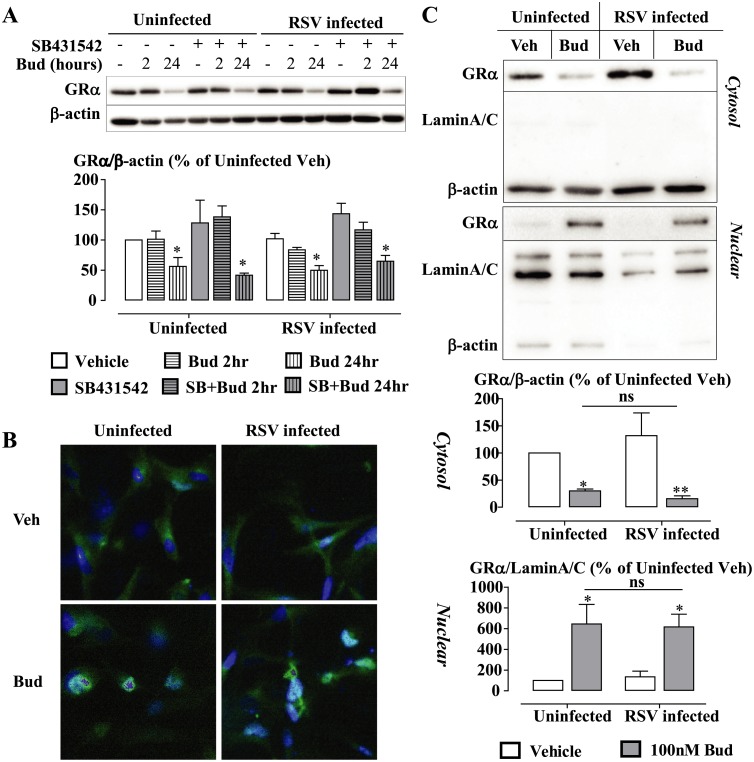
RSV infection does not impair GRα expression or nuclear localization. BEAS-2B cells were pre-incubated with/without SB431542 (1μM) for 30 min prior to RSV infection at MOI 0.1 for 48 h. Budesonide (Bud, 100nM) was added to the cells for the last 2 hours or last 24 hours in (A), and only for 2 hours in (B) and (C). **(A**) Cell lysates were analyzed by Western blotting. GRα expression was normalized to β-actin expression. **(B)** Cells were fixed with 10% NBF, then immunofluorescence of GRα was detected with a FITC-labelled secondary antibody, and nuclei were co-stained with DAPI. **(C)** Cells were extracted into cytosolic and nuclear fractions, and then subjected to SDS-PAGE and immunoblotted with anti-GRα. Matched proportions of the whole cell extract representing the nuclear and cytosolic fractions were loaded. The nuclear fraction is identified by expression of nuclear proteins, lamin A/C, which was not observed with the cytosolic fraction. Relative loading of the cytosolic fraction was established by β-actin levels. (A) and (C) images presented are representative of 3 independent experiments, and (B) Images presented are representative of 4 independent experiments. Data are presented as mean ± SEM of 3 independent experiments. *P<0.05, **P<0.01 significantly different from vehicle expression.

TGF-β-induced impairment of glucocorticoid action was partially attributed to attenuated nuclear translocation of GRα in the A549 cell line [47], although this was not observed in the BEAS-2B cell line [46]. We investigated the potential relevance of delayed or reduced GRα translocation, or changes in the level of GRα in RSV-infected BEAS-2B cells. Analysis of BEAS-2B cell cytoplasmic and nuclear extracts indicated that whilst SB431542 did not affect the total GRα protein expression level, 24 hours budesonide treatment (100nM) significantly reduced the expression of GRα protein (Fig 8A). However, neither the expression level nor the GRα subcellular distribution was influenced by RSV infection **(**Fig 8C). Immunoreactive-GRα was detected in both cytoplasmic and nuclear compartment in vehicle-treated cells. The immunoreactive-GRα level was increased in the nuclear compartment in response to budesonide treatment. However, the localization of GRα in the presence of budesonide was not affected by RSV infection (Fig 8B).

### RSV Infection-Induced Impairment of Glucocorticoid Action Is Prevented by the Clinically Used TGF-β Inhibitor, Tranilast

**Fig 9:**
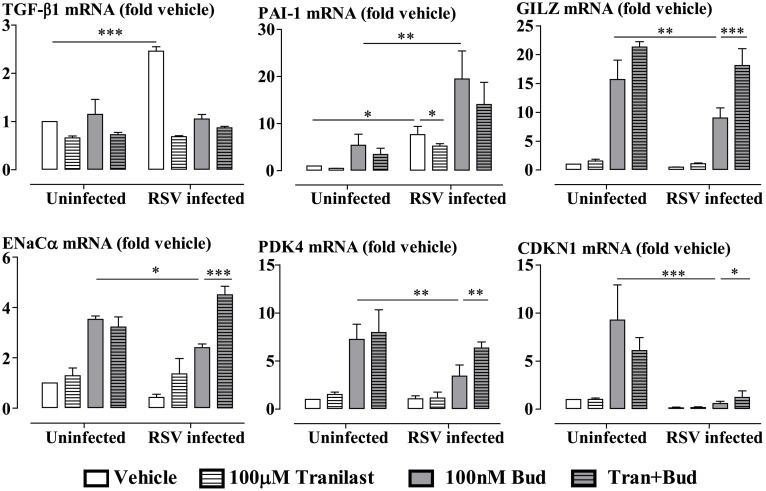
Tranilast prevents RSV impairment of budesonide-induced gene expression in BEAS-2B cells. The cells were pre-incubated with tranilast (100μM) for 30 min prior to 48 hours RSV infection. GC-inducible gene expression was assessed by stimulation with budesonide (Bud, 100nM) for the last 4 hours. Gene expression is expressed as fold change from the uninfected vehicle-treated cells. Data are presented as mean ± SEM, n = 3 independent experiments. *P<0.05, **P<0.01, ***P<0.001.

The anti-allergic agent tranilast inhibits the expression and activity of TGF-β in different cell types [50–52]. Importantly, this agent has few and only mild side-effects and is well tolerated [49]. We therefore examined the effects of tranilast at a concentration (100μM) within the range detected in plasma (30–300μM) after oral administration of a therapeutic dose [48], to ascertain its impact on GC impairment by RSV infection in epithelial cells. We found that tranilast inhibited RSV infection-induced mRNA expression of TGF-β1 and PAI-1 (Fig 9A). Pre-incubation of BEAS-2B cells with tranilast prevented/attenuated RSV infection impairment of budesonide-induced mRNA expression of GILZ, ENaCα, PDK4 and CDKN1C (Fig 9B), whilst it did not affect the intracellular expression of RSV A2 strain N gene (S3B Fig).

### Budesonide Does Not Affect RSV-Induced Anti-viral Cytokines, but Inhibits Pro-inflammatory Cytokine Production

**Fig 10:**
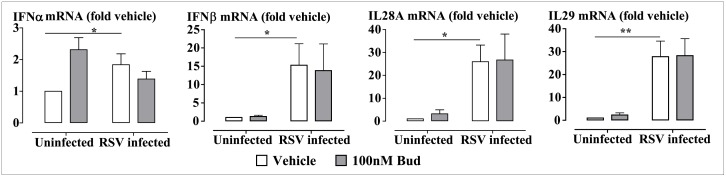
Budesonide does not affect RSV infection-induced expression of the anti-viral cytokines. BEAS-2B cells were infected with RSV at MOI 0.1 for 48 hours. Budesonide (Bud, 100nM) was added for the last 4 hours. Gene expression is plotted as fold change from the uninfected vehicle-treated cells. Data are presented as mean ± SEM, n = 4–5 independent experiments. *P<0.05, **P<0.01 significantly different from uninfected vehicle-treated cells.

Expression of the anti-viral cytokines IFN-α, IFN-β, IFN-λ1 (IL29) and IFN-λ2 (IL28A), and the pro-inflammatory cytokines IL-8 and IL-6 were measured. We found that RSV infection markedly induced the mRNA expression of IFN-β, IL29 and IL28A in BEAS-2B cells, whilst modest up-regulation of expression of IFN-α mRNA was observed. None of these expression levels were influenced by budesonide (Fig 10). RSV infection also induced marked expression of pro-inflammatory cytokines, including IL-8 and IL-6 mRNA in BEAS-2B cells. Treatment of the cells with budesonide (100nM) attenuated the RSV infection-induced expression of IL-8 and IL-6 mRNA by more than 80%. Inhibition of ALK5 using SB431542 (1μM) prior to RSV infection did not affect IL-8 expression. However, SB431542 (1μM) reduced the RSV infection-induced IL-6 expression by 30% (S8A Fig). Interestingly, pre-incubation of the cells with tranilast (100μM), a modulator of TGF-β production and activity, reduced RSV infection-mediated expression of both IL-8 and IL-6. Co-treatment with budesonide further inhibited IL-8 expression (S8B Fig)

### RSV Infection Impairs Glucocorticoid Transactivation in Differentiated Primary HBECs

**Fig 11:**
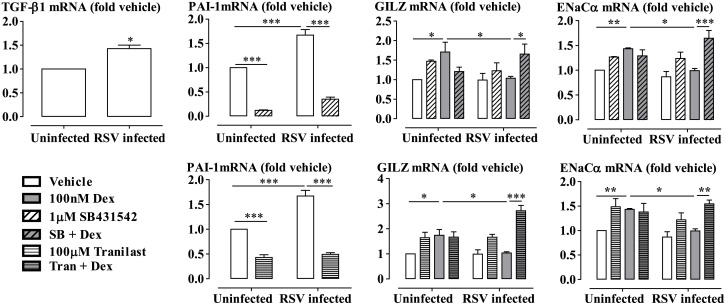
SB431542 or tranilast prevents RSV impairment of dexamethasone -induced gene expression in air-liquid-interface (ALI) differentiated primary human bronchial epithelial cells. Cells were pre-incubated with SB431542 (SB) or tranilast (Tran) for 1 hour prior to RSV infection at MOI 0.1 for 48 hours. Dexamethasone (Dex, 100nM) was added for the last 5 hours. The levels of gene expression are expressed as fold change from the uninfected vehicle. Data are presented as mean ± SEM, n = 3 primary cultures. *P<0.05, **P<0.01, ***P<0.001.

Primary HBECs were cultured at air-liquid interface (ALI-HBECs) to reach the criteria for ALI differentiation, including TEER values of at least 200 Ω.cm^2^ to ascertain the formation of functional tight junctions; increased mRNA expression of Tektin-1 (a marker for ciliated cells) and MUC5AC (a marker of goblet cells); and visible cilia and mucus on the differentiated cells [46]. RSV infection increased expression of TGF-β1 and the TGF-β-inducible gene PAI-1 mRNA (Fig 11). The PAI-1 expression was reduced by pre-incubation of the cells with SB431542 (1μM) or tranilast (100μM) for 1 hour prior to RSV infection. RSV infection impaired the dexamethasone-induced mRNA expression of GILZ and ENaCα. Importantly, the impairment of the expression of the genes was prevented by SB431542 or tranilast (Fig 11).

## Discussion

**Fig 12:**
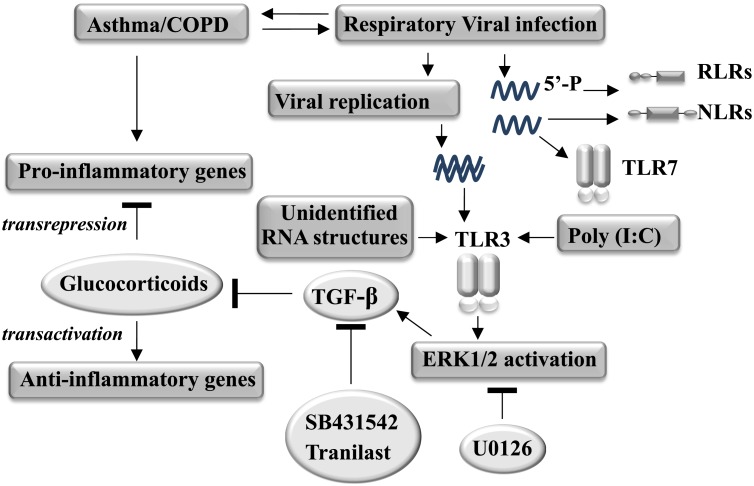
Proposed mechanism of viral infection-induced glucocorticoid- insensitivity. The respiratory viral infection or Poly(I:C) stimulation activates the TLR3-ERK1/2 pathway, to induce TGF-β activity, which is a key mediator of the GC impairment. Modulation of TGF-β expression or activity is a potential strategy for restoring GC sensitivity during the respiratory viral infection.

Respiratory viral infection-induced acute bronchiolitis and asthma/COPD exacerbations are worldwide health problems, with a substantial disease burden in the young, the elderly, in adults with chronic lung disease and patients who are immunocompromised [3, 4]. Inhaled or oral glucocorticoids are standard treatments for asthma and COPD, but GCs are generally not effective for treating exacerbations of asthma and COPD, and other inflammatory complications of respiratory virus infection. In this study, we identified that endogenous TGF-β is expressed, induced TGF-β-like activity, increases PAI-1 expression in RSV, RV or IAV-infected bronchial epithelial cells, contributing to the viral infection-induced GC insensitivity. We also showed that treatment of epithelial cells with the anti-allergic agent tranilast reduced the expression and activity of TGF-β, and restored GC sensitivity (Fig 12).

RSV infection impaired GRE activity and the expression of GC targeted genes, including GILZ and ENaCα in the BEAS-2B bronchial epithelial cell line. GILZ is a GC-responsive gene that mediates anti-inflammatory effects of GCs in T cells, macrophages and also epithelial cells [57]. Impairment of GILZ expression by viral infection dampens the anti-inflammatory activity of GCs. ENaC channels in lung epithelial cells regulate the airway surface fluid levels. The attenuation of ENaCα expression by viral infection leads to excess fluid and recurrent infections in the lung [58]. As the primary ALI-HBECs are necessarily cultured in hydrocortisone-containing medium, the response to synthetic GC stimulation is modest. Nevertheless, RSV infection-induced GC activity impairment was also observed in ALI-HBECs. Importantly, we found that RSV infection-impaired expression of GC-responsive gene (ENaCα and PLZF) translates to similar patterns of change in protein levels. This impairment of GC action may explain the lack of clinical effectiveness of GC treatment in RSV-infected patients. Interestingly, we found that RV and IAV also induced TGF-β-like activity, observing increased PAI-1 expression and TGF-β-dependent impairment of GC activity in BEAS-2B cells. Thus, GC resistance is likely a common response to a respiratory viral infection.

The viral pathogen-associated molecular patterns are detected by pattern recognition receptors, including TLRs, RLRs and NLRs, expressed in or on respiratory epithelial cells. We found that GC impairment was also induced by the TLR3 agonist, poly(I:C), a synthetic analog of dsRNA. Poly(I:C) stimulation also activates RLRs (RIG-1 and MDA-5). However, activation of RLRs with Poly(I:C)HMW/LyoVec did not affect the GC actions. RSV and IAV comprise negative-sense ssRNA genome viruses, classified as a paramyxovirus and orthomyxovirus, respectively. RV is classified as a picornavirus and has a positive-sense ssRNA. RSV and RV generate dsRNA intermediates during viral replication cycles, which activate TLR3 [59–61]. IAV does not generate abundant levels of dsRNA in the infected cells. However, TLR3 is thought to recognize as yet unidentified RNA structures during IAV infection [13]. In order to validate that impairment of GC action with each of these viruses is through activation of TLR3 in the infected cells, we chose to knockdown TLR3 using targeted siRNA. Whilst TLR3 expression was reduced approximate 70%, the viral infection-induced TGF-β expression and GC impairment were prevented. These data strongly suggest that viral infection-impaired GC action is at least partially mediated by activation of TLR3 (Fig 12).

Engagement of TLR3 activates multiple transcription factors including NF-κB, mitogen-activated protein kinases (MAPKs), and members of the interferon regulatory factor (IRF) family, which induce the expression of inflammatory cytokines and type I/III IFNs. Since both viral infection and poly(I:C) stimulation induces secretion of various cytokines [62], it is conceivable that the GC impairment was mediated by the release of soluble factors that act in an autocrine manner. Some of these cytokines (such as TNFα, IFNγ, IL4, IL13 and IL17) have been reported to interfere with GC action in epithelial cells [38–40]. Viral infection induces expression and secretion of TGF-β in epithelial cells [41, 42]. Our group has recently shown that TGF-β impairs GRE-dependent transactivation in different epithelial cell types [46, 47]. Moreover, we found that TGF-β was more potent, had a more rapid onset and shows a greater extent of GC impairment than the combination of TNFα, IL-4 and IL-13. We now show that RSV infection induces TGF-β expression and activity in the BEAS-2B cell line and ALI-HBECs. Moreover, we found that RSV infection-induced TGF-β expression was mediated by phosphorylation of ERK1/2, at least partially through activation of TLR3. Inhibition of ERK1/2 activation significantly attenuated the impairment of GC activity by RSV. Inhibition of ALK5 with SB431542 tended to reduce the phosphorylation of ERK1/2. However, we have shown previously that TGF-β-induced GC impairment was not induced by activation of ERK nor other established canonical or non-canonical pathways. A novel TGF-β-inducible mechanism is implicated in the modulation of GC action [46]. Therefore, we believe the contribution of ERK1/2 activation to the viral infection-induced GC action occurs upstream of TGF-β expression, in signaling emerging from activation of TLR3 (Fig 12).

Viral infection induces cytopathic effects reducing cell viability. The cytopathic effect is both time and inoculation dose (MOI) dependent. RSV infection decreased the cell numbers compared to the uninfected cells. However, under the conditions of RSV infection in the present study there were no detectable effects on cell viability, suggesting that viral induced GC impairment is not secondary to reduced cell viability. We found that inhibition of the type I TGF-β receptor ALK5 activity did not impact the viral reduction in cell numbers or viral replication. Interestingly, inhibition of ALK5 activity prevented/attenuated the RSV-impaired GRE activity and the expression of its targeted genes, including GILZ and ENaCα, which suggests that blockade of TGF-β activity increases the GC-mediated anti-inflammatory action and airway fluid regulation. Viral infection induced activation of the TGF-β/ALK5 pathway and subsequent impairment of GC action was further confirmed by knockdown of ALK5 using targeted siRNA. Thus, autocrine TGF-β contributes to the viral infection-induced GC insensitivity in airway epithelial cells, identifying TGF-β signaling as a target for inhibition that can potentially restore GC sensitivity during RSV infection (Fig 12). Moreover, we found that poly(I:C) or viral infection-impaired GC activity was shown after 24–48 hours incubation or infection. A similar latency period has been reported by another group showing that poly(I:C) or RV infection-decreased GC activity only became apparent after a period of hours and reached maximum in 24–48 hours post-treatment [36]. The incubation period fits our conclusion with regard to the time required for dsRNA generation by viral replication, TLR3 activation-induced TGF-β expression and activity. We found that RSV infection also reduced on-going responses to budesonide. This latter experimental design is of relevance to therapeutic patterns in asthma and COPD and offers a potential explanation of the exacerbations upon respiratory viral infection. We suggest that viral infections not only induce inflammatory pathways that are intrinsically insensitive to GC, but also that the asthmatic or COPD inflammation previously controlled by GC is compromised by infection induced TGF-β activity.

RSV infection impaired most of the GC-responsive genes assessed. However, we also found GCs might have beneficial effects in regulation of RSV-induced cytokine expression, as budesonide inhibited RSV-induced mRNA encoding the pro-inflammatory cytokines IL-8 and IL-6, without interfering with the production of IFNs or viral replication. The mechanism of tranilast-inhibition of TGF-β production and activity was unclear. It is likely acting differently from SB431542, but has in common with SB431542 suppression of TGF-β expression and activity. Interestingly, we have shown for the first time that tranilast, but not SB431542, markedly inhibited the RSV-induced expression of IL-8 and IL-6, which suggests the potential for additional beneficial anti-inflammatory activities mediated by tranilast, when used as an anti-allergic agent.

The molecular mechanisms of TGF-β impairment of GC-action in epithelial cells have been extensively studied [46, 47]. The impairment was unrelatedto either the GRα protein level, or to the GRα nuclear translocation in BEAS-2B cells. The GC impairment by TGF-β requires activation of ALK5. However, the signal transduction downstream of ALK5 could not be associated with any known canonical or non-canonical pathways [46, 47]. Similar results have been found with RSV infection-impaired GC action that GRα protein expression or GRα nuclear translocation were not influenced by RSV infection. Moreover, inhibition of ALK5 did not affect the expression of GRα protein level. Current evidence suggests a novel non-canonical signaling pathway being activated. Hypothesis-free approaches, such as proteomics and functional genomics, are being used to further examine the signaling mechanisms subserving GC resistance induced by TGF-β [29].

TGF-β activates a variety of signaling cascades regulating many cellular processes. Global inhibition of TGF-β activity therefore engenders many adverse effects, including excessive inflammation and risk of autoimmunity [56, 63]. Further investigation of the novel signaling mechanism underlying the GC impairment by TGF-β and its more selective targeting may restore GC sensitivity during respiratory viral infection, whilst avoiding the adverse effects that are associated with complete inhibition of TGF-β activity. TGF-β modulators may be an alternative means to restore GC activity without undue adverse effects. The anti-allergic agent tranilast is reported to inhibit the expression and activity of TGF-β in different cell types [50–52]. Importantly, it has few and only mild side-effects and is well tolerated [49]. We found that tranilast inhibits the expression and activity of TGF-β in both BEAS-2B cells and ALI-HBECs. Intriguingly, we show that pre-incubation with tranilast prevented the GC impairment by RSV infection. Further establishing the effectiveness of tranilast in viral infection would support the use of TGF-β modulators for the prevention/treatment of GC insensitivity occurring during RSV infection-induced bronchiolitis or asthma/ COPD exacerbations.

In summary, exacerbations of asthma or COPD associated with respiratory viral infection are resistant to the anti-inflammatory actions of GCs. We identified autocrine TGF-β as a key mediator of the GC impairment. Our studies show for the first time that modulation of TGF-β activity is a potential strategy for restoring the GC sensitivity during viral infection and for prevention of viral exacerbation of chronic airway diseases.

## Methods

### Cell Culture

BEAS-2B bronchial epithelial cells (ATCC, Manassas, VA, USA) were cultured as described [47], seeded at 5×10^4^ cells/cm^2^ in 24 well plates, T-75 flask or chamber slides in Dulbecco’s modified Eagle’s media (DMEM) containing 5% vv^-1^ heat-inactivated FBS, 15 mM HEPES, 0.2% vv^-1^ sodium bicarbonate, 2 mM L-glutamine, 1% vv^-1^ non-essential amino acids, 1% vv^-1^ sodium pyruvate, 5 IU·mL^-1^ penicillin and 50 mg·mL^-1^ streptomycin, and incubated overnight at 37°C in air containing 5% CO_2_. The cells were then inoculated with RSV at a multiplicity of infection (MOI) of 0.1 TCID50 (50% tissue culture infectious dose) per cell for 1 hour, and incubated for up to 48 hours. The GC transactivation was assessed by incubating the cells with budesonide (Bud, 0.01-100nM) for the last 24 hours to measure the glucocorticoid response element (GRE) activity, or for the final 4 hours, to measure the mRNA expression of the GC-inducible genes. In some experiments, budesonide was added to BEAS-2B cells for 24 hours or 4 hours prior to RSV infection and it was re-added after 1 hour RSV inoculation. The mRNA expression of the GC-inducible genes and also protein were examined after RSV infection for up to 48 hours.

### Air-Liquid Interface Differentiation of Primary HBECs

Primary HBECs were purchased from Lonza (Waverley, Australia) and cultured using B-ALI Bulletkit (Lonza) according to the manufacturer’s instructions. The cells were differentiated for more than 21 days at air-liquid interface on fibrillar collagen-coated 24-well Corning Transwell 0.4μm pore polyester membrane cell culture inserts (Sigma-Aldrich, MO,USA) as described [46]. Cell differentiation was confirmed through measurement of trans-epithelial electrical resistance (TEER) and visualization of beating cilia. RSV at a MOI of 0.1 or control culture medium was added onto the apical surface of the cells, which were inoculated for 1 hour, and then incubated for up to 48 hours. Dexamethasone (Dex, 100nM) was applied to the basolateral side of the cells for 5 hours to assess the GC transactivation by measuring the mRNA expression of the GC-inducible genes.

### Virus Stock Preparation and Infection

Human RSV, prototype A2 strain (ATCC VR-1540) was cultured in Hep2 cells (also from the ATCC). Viruses were inoculated into Hep2 cells, and incubation continued until a cytopathic effect was observed. Supernatant was removed and the Sucrose-Phosphate-Glutamate-Albumin (SPGA) stabilizer solution was added to the cells. The virus was harvested by scraping the cells and centrifugation the cell suspension at 1,000g for 15 min. clarified supernatants were snap frozen and stored at -80°C RSV was titrated by serially diluting the newly generated stock virus (1/10^3^−1/10^8^) and then inoculating Hep2 cells in flat-bottomed 96-well plates (2.5×10^4^ cells/well). Viral titer was determined by TCID50 assay, defined as the quantity of virus which induces detectable cytopathic effects in 50% of the infected cells after 3–5 days, and was calculated according to Reed and Muench [64].

Human rhinovirus, RV16 strain (ATCC VR-283) was cultured in Ohio-HeLa cells (a kind gift from Dr. Reena Ghildyal). The virus was harvested by scraping the cells without removing the infection media. The cell suspension was centrifuged at 3,000g for 15 min. The viral titer was titrated by using the same methodology as RSV, but in Ohio-Hela cells.

Influenza A virus, HKx31 strain (also known as X-31, a virus strain of H3N2 subtype, a kind gift from Dr. Sarah L. Londrigan) was cultured in the allantoic cavity of 10-day old embryonated chicken eggs (Research Poultry, Research, Victoria, Australia) and titrated on Madin-Darby canine kidney (MDCK) cells (ATCC) by standard procedures and expressed as plaque forming units (PFU)/ml as previously described [65].

### Cell Transfection

BEAS-2B cells for transfection were seeded in 24-well plates overnight. Cells were co-transfected with pGRE-SEAP and pGL3 control plasmids using Lipofectamine 2000 (Invitrogen, Carlsbad, CA), as previously described [46, 47]. Transfected cells were inoculated with RSV at a MOI of 0.1 or control medium for 1 hour, and incubated for 24 hours prior to the addition of Bud (1 nM) or vehicle for further 24 hours. The 24 hour time point for Bud-induced GRE activity was selected based on our previous studies [46, 47]. Supernatants were collected for measurement of secreted SEAP using a chemiluminescence kit (Roche Applied Science, NSW, Australia) as described [46]. Pre-validated siRNA targeting ALK5 and TLR3 (Invitrogen) was transfected using Lipofectamine RNAiMAX (Invitrogen) as described previously [66].

### Western Blot Analysis

BEAS-2B cells were seeded in 6-well plates overnight. Cells were pre-incubated with SB431542 (1μM) for 30 min prior to RSV infection at MOI of 0.1 or control medium for 3 hours, 24 hours and 48 hours for assessment of intracellular kinase phosphorylation. To assess changes in total GRα, ENaCα and PLZF expression, budesonide (100nM) was added to the cells following 48 hours RSV infection (MOI 0.1) for the last 2 hours or the last 24 hours. In some experiments, PLZF expression was measured after treatment of the cells with budesonide for 4 hours prior to RSV infection for 48 hours. Rabbit polyclonal antibody (pAb) anti-phospho-ERK1/2 (Thr202/Tyr205) and rabbit monoclonal antibody (mAb) anti-Erk1/2 (Cell Signaling) was used to measure the ERK1/2 activation. Rabbit pAb anti-GRα (Santa Cruz Biotechnology) was used to measure GRα expression. Mouse monoclonal antibody anti-PLZF and goat polyclonal antibody anti-αENaC (Santa Cruz Biotechnology) were used to measure the expression of PLZF and ENaCα. The expression level of GAPDH protein (Rabbit pAb; Abcam, Cambridge, UK) was used as a reference control for normalization to account for variation in protein loading. Western blotting was performed as described [66]. Band intensities were quantified by densitometry using the image J program (1.48v, National Institute of Health, USA).

### GRα Localization Analysis

BEAS-2B cells were seeded in a T-75 flask for isolation of cytosolic and nuclear fractions, or in an 8 chamber slide for immunofluorescence staining. Cells were infected with RSV at MOI of 0.1 or control culture medium for 46 hours prior to addition of Bud (100nM) for 2 hours. GRα localization was then determined by subcellular fractionation followed by western blot analysis as described [47]. In separate experiments, immunofluorescence was used to monitor GRα localization with the DAPI-stained nucleus [47].

### Cell Viability

Cell viability was assessed using the Trypan blue exclusion method, as described [66].

### RNA Extraction and Real Time Quantitative PCR (RT-qPCR)

BEAS-2B cells were seeded in 24-well plates overnight. Cells were pre-incubated with SB431542 (1μM) for 30 min prior to RSV infection at MOI of 0.1, RV infection at MOI of 1, IAV infection at MOI of 0.1, or control medium for 44 hours prior to addition of Bud (0.01-100nM) or Dex (30nM) for 4 hours. The 4 hour time point for mRNA expression of the GC-inducible genes was chosen based on our previous studies [46, 47]. In some experiments, tranilast (100μM) or U0126 (1μM) was added 30 min prior to RSV infection. Cells were also treated with TLR3 agonist, polyinosinic-polycytidylic acid (poly(I:C); 10μg/ml); or RLRs ligands, Poly(I:C)(HMW)/LyoVec (0.01–1μg/ml) for 24 hours prior to addition of Dex (30nM) for 4 hours. The mRNA extraction and reverse transcription were performed as previously described [46]. An ABI Prism 7900HT sequence detection system (Applied Biosystems) was used to quantitatively analyze the level of gene expression as previously described [47]. The generation of specific PCR products was confirmed by dissociation curve analysis. 18S ribosomal RNA (18S rRNA) was used as a housekeeping gene. RSV N gene mRNA expression level was determined by the standard curve on the basis of known TCID 50 virus stock. Primer sequences (Table 1) were KiCqStart pre-designed primers from Sigma-Aldrich, or obtained from the literature, or designed using Primer Express software (Applied Biosystems, Mulgrave, Australia) with mRNA sequences from the National Centre for Biotechnology Information (http://www.ncbi.nlm.nih.gov).

**Table 1:** Primer Sequences for RT-qPCR.

Genes	Primer sequences (5’ to 3’)
18srRNA [67]	FP: CGCCGCTAGAGGTGAAATC
	RP: TTGGCAAATGCTTTCGCTC
GILZ [57]	FP: TCCTGTCTGAGCCCTGAAGAG
	RP: AGCCACTTACACCGCAGAAC
ENaCα [68]	FP: AGCACAACCGCATGAAGAC
	RP: TGAGGTTGATGTTGAGGCTG
SERPINA3*	FP:TCAAGACAAGATGGAGGAAG
	RP:TCACCTATCTCTCTGAACTC
CDKN1C*	FP:TCTGATCTCCGATTTCTTCG
	RP:CTCTTTGGGCTCTAAATTGG
PDK4*	FP:CTTGGGAAAAGAAGACCTTAC
	RP:GTGCAGTGGAGTATGTATAAC
KCNB1*	FP:AAGATCCTTGCCATAATTTCC
	RP:GAACCTCAGCAGGTACTC
PLZF*	FP:GTTCCTGGATAGTTTGCG
	RP:CATGTCAGTGCCAGTATG
TGFB1 [69]	FP: CCCAGCATCTGCAAAGCTC
	RP: GTCAATGTACAGCTGCCGCA
PAI-1^§^	FP: TCAGGCTGACTTCACGAGTCTTT
	RP: CTGCGCGACGTGGAGAG
IL-8 [70]	FP: CTGGCCGTGGCTCTCTTG
	RP: CCTTGGCAAAACTGCACCTT
IL-6 [71]	FP: AGCTCTATCTCGCCTCCAGGA
	RP: CGCTTGTGGAGAAGGAGTTCA
IFN-α [72]	FP:GTGAGGAAATACTTCCAAAGAATCAC
	RP:TCTCATGATTTCTGCTCTGACAA
IFN-β [73]	FP:CAGCAATTTTCAGTGTCAGAAGC
	RP:TCATCCTGTCCTTGAGGCAGT
IL28A*	FP: ACATAGCCCAGTTCAAGTC
	RP: GACTCTTCTAAGGCATCTTTG
IL29*	FP: CAGGTTCAAATCTCTGTCAC
	RP: AACTCCAGTTTTTCAGCTTG
RSV N gene [74]	FP:GCTCTTAGCAAAGTCAAGTTGAATGA
	RP:TGCTCCGTTGGATGGTGTATT

*KiCqStart pre-designed primers

^**§**^ Designed primers using Primer Express software

### Statistical Analyses

Data are expressed as the mean ± SEM. Reported *n* values represent number of experiments repeated or number of primary cultures used. All data were statistically analyzed using GraphPad Prism 5.0 for Windows (GraphPad Software, San Diego, CA). One-way or two-way analyses of variance (ANOVA) with Bonferroni’s *post hoc* test were used to analyze the data. A *P* value less than 0.05 was considered statistically significant.

## Supporting Information

S1 Fig RSV infection impaired budesonide-induced GRE activity is prevented by pre-incubation with SB431542 (1μM). BEAS-2B cells were pre-incubated with SB431542 (1μM) for 30 min prior to RSV infection at MOI 0.1 for 48 hours. Budesonide (Bud, 1nM) was added for the last 4 hours of the RSV infection. The level of SEAP in the supernatants was expressed as a percentage of the level induced in response to Bud treatment in the uninfected group, Data are presented as mean ± SEM, n = 4 independent experiments,***P<0.001.
(EPS)

S2 Fig RSV infection-reduced cells number was not due to cell death (A), and bot affected by ALK5 inhibition with SB431542 (B). RSV infection for 48 hours at MOI 0.1 decreases the total cell number, but has no detectable effects on cell viability compared to the uninfected cells (**A**, n = 9). Pre-incubation of the cells with 1μM SB431542 does not impact the cell numbers (**B**, n-3). RSV infection Data are presented as mean ± SEM, n = 3–9 independent experiments, *P<0.05, **P<0.01, ***P<0.001.
(EPS)

S3 Fig The intracellular titer of RSV was not influenced by treating the cells with ALK5 inhibitor SB431542 or tranilast, or treating the cells with budesonide. BEAS-2B cells were pre-incubated with SB431542 (1μM) (**A**, n = 6) or tranilast (100μM) (**B**, n = 3) for 30 min prior to RSV infection at MOI 0.1 for 48 hours. Budesonide (Bud, 100nM) was added for the last 4 hours. RSV N gene mRNA expression was determined by RT-qPCR. Data are presented as mean ± SEM, n = 3–6 independent experiments.
(EPS)

S4 Fig Potent TGF-β type I receptor (ALK5) inhibitor GW788388 prevents RSV impairment of budesonide-induced transactivation. BEAS-2B cells were incubated with GW788388 (1μM) for 30 min prior to RSV infection at MOI 0.1 for 48 hours. Budesonide (Bud) was added for the last 4 hours. Total RNA was extracted and gene expression was measured by RT-qPCR. Gene expression is expressed as fold change from uninfected vehicle-treated cells. Data are presented as mean ± SEM, n = 3. *P<0.05, **P<0.01, ***P<0.001.
(EPS)

S5 Fig Knockdown of ALK5 increased budesonide-induced gene expression in RSV infected cells (C). BEAS-2B cells were transfected with scrambled control or ALK5-targeted siRNA, then infected with RSV (MOI 0.1) for 20 hours, followed by stimulation with Bud (100nM) for 4 hours. **(A)** ALK5 siRNA reduced ALK5 expression. **(B)** Transfected BEAS-2B cells were incubated with TGF-β (40pM) for 24 hours. TGF-β-induced phosphorylation of Smad2 reduced in the ALK5 siRNA transfected cells compared with the control siRNA transfected cells. Data are presented as mean ± for n = 3–4 independent experiments. **P<0.01, **P<0.001.
(EPS)

S6 Fig Activation of RLRs with Poly(I:C)HMW/LyoVec did not affect the GC actions. BEAS-2B cells were treated with RIG-1/MDA5 ligands, poly(I:C)/LyoVec complexes for 20 hours, followed by stimulation with dexamethasone (Dex, 30nM) for 4 hours. Data are presented as mean ±SEM for n = 4 independent experiments.
(EPS)

S7 Fig Poly(I:C) stimulation induces ERK1/2 activation. BEAS-2B cells were treated with Poly(I:C) (10μg/ml) for 2 hours, 6 hours and 24 hours. Expression of phosphorylation of ERK1/2 (p-ERK1/2) was analyzed by Western blotting. The membrane was stripped and re-probed with total Erk1/2 (t-ERK1/2), and then stripped again and re-probed for GAPDH expression for normalization. Protein expression level was expressed as a percentage of vehicle groups. Densitometry data are presented as mean ± SEM of 4 experiments. *P<0.05, Poly(I:C) *c*.*f*. vehicle.
(EPS)

S8 Fig Budesonide inhibits RSV infection-induced expression of the pro-inflammatory cytokines. BEAS-2B cells were treated with SB431542 (1μM) **(a)**, tranilast (100μM) **(b)** or vehicle for 30 min prior to RSV infection at MOI 0.1 for 48 hours. Budesonide (Bud, 100nM) was added for the last 4 hours. The gene expression level is expressed as fold change from the uninfected vehicle-treated cells. Data are presented as mean ± SEM, n = 3–5 independent experiments. ***P<0.001.
(EPS)
